# Thermal Oxidation Gas-Release Strategy for Scalable Synthesis of Porous SnO_2_ Towards High-Performance Supercapacitor

**DOI:** 10.3390/gels12060476

**Published:** 2026-05-29

**Authors:** Xiaoli Wang, Xinyu Zhao

**Affiliations:** 1 Liaoning Provincial Key Laboratory of Energy Storage and Utilization, College of Chemistry and Environment Engineering, Yingkou Institute of Technology, Yingkou 115014, China; wangxl@yku.edu.cn; 2 College of Chemistry and Materials Science, Inner Mongolia Minzu University, Tongliao 028000, China; 3 National Demonstration Center for Experimental Chemical Education, Inner Mongolia Minzu University, Tongliao 028000, China

**Keywords:** supercapacitor, SnO_2_, porous materials, gel

## Abstract

Conventional strategies for synthesizing porous structures generally depend on template-based methods, which involve not only excessive consumption of templating agents but also the use of hazardous chemicals, such as hydrofluoric acid or strong alkalis. Therefore, designing an effective and convenient strategy to fabricate porous SnO_2_ is of significant practical relevance. Herein, we developed a top-down strategy to fabricate SnO_2_ electrode via a thermal oxidation gas-release route, resulting in a bulk 3D hierarchical architecture with interconnected porous channels. Employing a bottom-up strategy, the gel precursors of these porous SnO_2_ materials were synthesized on a large scale via a simple, surfactant- and template-free route, in accordance with green chemistry principles. The results show that the porous SnO_2_(300) electrode materials possess a high specific surface area and exhibit favorable electrochemical energy-storage performance, achieving a high specific capacitance of 267.31 F g^−1^ at a current density of 1 A g^−1^. Furthermore, based on the gel electrolyte of PVA/KOH, an asymmetric supercapacitor device assembled using porous SnO_2_(300) materials as the positive electrode and activated carbon as the negative electrode (denoted as P-SnO_2_//AC) achieves an energy density of 32.49 Wh kg^−1^ at the power density of 718.97 W kg^−1^. This work presents a simple, cost-effective, environmentally friendly and scalable approach to synthesize SnO_2_ materials with an advanced structural design.

## 1. Introduction

In light of the escalating depletion of fossil energy resources and the increasing frequency of extreme climatic events, the demand for green, renewable and clean energy sources has grown significantly in recent years [1,2]. Meanwhile, driven by the global dual-carbon goals of “carbon peak” and “carbon neutrality”, sustainable energy systems such as solar, wind and ocean power have undergone extensive development and deployment to reduce environmental and energy crises [3,4]. Consequently, given the inherent intermittency and variability of these sources, which impede their large-scale integration, the development of cost-effective, flexible, and efficient energy-storage devices is essential for transitioning to a low-carbon society. Among alternative energy-storage technologies, supercapacitors (SCs) are considered highly promising candidates for next-generation electrochemical energy-storage devices. This arises from their superior high-power density, enhanced charge–discharge rates, and exceptional cycling stability relative to conventional energy-storage systems such as batteries and traditional capacitors [5,6,7]. These advantages have positioned supercapacitors as increasingly prominent candidates in the field of advanced energy research. Furthermore, the utilization of aqueous electrolytes in supercapacitors offers enhanced environmental compatibility and non-flammability. Based on their charge-storage mechanisms, supercapacitors are categorized as electric double-layer capacitors (EDLCs-type), pseudo-capacitors (PCs-type) and hybrid supercapacitors [8,9,10,11]. EDLCs-type electrodes are typically constructed from carbon-based materials, such as carbon nanotubes, graphene and activated carbons, that store energy via non-faradaic processes, including physical adsorption/desorption of ions or molecules on the surface of electrode [12,13,14]. However, the widespread application of EDLCs-type in energy-storage remains constrained by their relatively low-energy density. In contrast, PCs-type, which store charges through faradaic redox reversible reactions, have attracted significant research interest owing to their potential in achieving higher-energy density and specific capacitance [15,16,17]. As research advances, PCs-type materials have become increasingly diverse, exhibiting distinct charge–discharge profiles governed by their specific charge-storage mechanisms, which encompass surface redox reactions, ion intercalation, and battery-type intercalation/redox processes [9,17,18]. Hybrid supercapacitors (HSCs) integrate the high energy density of pseudo-capacitive or battery-type electrodes with the high-power density and long cycle life of EDLCs-type, thereby achieving a balanced energy-storage performance [17,18,19].

To date, various electrode materials such as conducting polymers, transition metal sulfides and metal oxides have been developed to improve performance of supercapacitors [20,21,22,23,24,25]. Within the landscape of electroactive candidates, metal oxide, particularly SnO_2_, has been explored as a promising material for energy-storage devices, since it exhibits high theoretical specific capacitance, excellent electrochemical activity, low cost, chemical durability and natural abundance [26,27]. However, the capacitive performance of conventional bulk SnO_2_ is still constrained, mainly owing to its relatively low specific surface area and insufficient electrochemically active sites [28]. Given the pivotal role of fabrication techniques in governing supercapacitor performance, enhancing capacitance through rational structural design has been recognized as an essential strategy [29,30,31]. Recent progress in the fabrication of electrode materials has led to significant improvements in supercapacitor performance. Considerable research efforts have been devoted over the past few decades to the synthesis of SnO_2_-based materials. Zhang et al. fabricated three-dimensional SnO_2_ with lamellar nanostructures directly on a carbon cloth substrate; the resulting electrode delivered a high specific capacitance of 247 F g^−1^ at a current density of 1 A g^−1^ when evaluated as an electrode material for supercapacitors [32]. Employing a facile hydrothermal approach, Guo et al. synthesized a porous SnO_2_/reduced graphene oxide composite, which exhibited a specific capacitance of 241 F g^−1^ at a current density of 1 A g^−1^ [33]. Through a solvothermal method followed by post-annealing in air, Luo et al. synthesized mesoporous SnO_2_ materials possessing high specific surface areas of 103.48 m^2^ g^−1^, which displayed battery-type electrochemical response with a maximum specific capacity of 219.3 C g^−1^ at 1 A g^−1^ [34]. Zarei et al. developed a cold spray technique to produce porous films containing abundant nanochannels, which delivered a specific capacitance of 0.0176 F cm^−2^ at a current density of 0.1 mA cm^−2^ [35]. Notwithstanding these achievements, the widespread commercial application of SnO_2_-based electrodes continues to be impeded by limitations including low-yield, intricate fabrication procedures, complex reaction conditions, and high cost. Consequently, developing SnO_2_ electrodes that integrate a low-cost and simple synthesis process remains a considerable challenge for scalable manufacturing and real-world implementation.

Recently, porous materials have garnered considerable interest as promising supercapacitor electrodes due to their 3D spatial architectures, which provide extra active sites and high specific surface areas, thereby promoting efficient ion and electron transport [30]. Nevertheless, conventional strategies for fabricating such porous structures generally rely on removable or sacrificial templates, often introducing complexity and additional cost [36,37,38,39,40,41]. To achieve the wide commercial application of supercapacitors, it is necessary to increase their energy density while still reducing their manufacturing costs. This highlights the urgent need to develop efficient and straightforward template-free strategies for synthesizing uniquely porous SnO_2_ materials, which hold great promise for next-generation supercapacitors. Recently, gas-evolution strategies have been demonstrated as effective routes for creating porous structures [42,43]. For example, Al-Gethami et al. utilized urea as an external fuel to generate gases during high-temperature combustion, which effectively created abundant pores in the synthesized Nd_2_O_3_-V_2_O_5_ nanocomposites [43]. Inspired by this concept, we developed a unique in situ gas-release strategy based on the thermal decomposition of a tin oxalate precursor. Unlike external-fuel-assisted combustion, our method generates gases intrinsically from the precursor itself, without any additives. This in situ process yields a hierarchical porous SnO_2_ structure with high specific surface area, while avoiding the need for templates or complex post-treatment.

With the above considerations, herein, uniform bulk SnO_2_ materials featuring a porous architecture were synthesized on a large-scale as a supercapacitor electrode via a simple thermal oxidation gas-release method. The resultant porous structure offers a high specific surface area, abundant redox-active sites and efficient ion-transport pathways through optimization of sintering temperature, which collectively contribute to compelling electrochemical energy-storage performance. This is evidenced by a high specific capacitance of 267.31 F g^−1^ at 1 A g^−1^ and remarkable cyclic stability, retaining excellent performance over 5000 cycles at 1 A g^−1^. This synthesis method, characterized by simplicity, cost-effectiveness, and scalability, provides significant advantages for the fabrication of porous materials with high surface area. We anticipate that this strategy can be extended to synthesize other advanced transition metal oxides for applications beyond energy storage, such as in catalysis or gas sensors.

## 2. Results and Discussion

**Figure 1:**
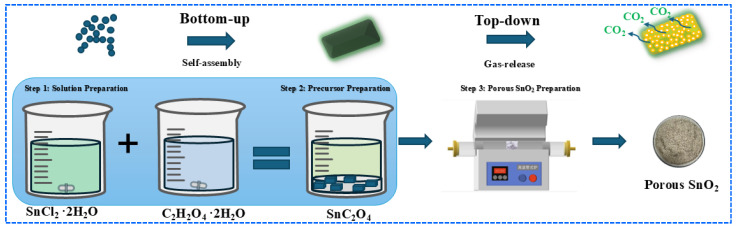
A schematic diagram of the synthetic procedure of porous SnO_2_ materials.

The schematic diagram of the overall preparation process for SnO_2_ electrode materials with porous architecture is illustrated in Figure 1. In a typical synthesis, the precursor of porous SnO_2_ materials is synthesized via a facile, surfactant- and template-free precipitation method, utilizing a bottom-up technique at room temperature. In this reaction, stannous chloride dihydrate (SnCl_2_·2H_2_O) and oxalic acid dihydrate (H_2_C_2_O_4_·2H_2_O) were employed as the tin source and precipitating agent, respectively. H_2_C_2_O_4_·2H_2_O undergoes dissociation, liberating oxalate ions (C_2_O_4_^2−^) that subsequently complex with Sn^2+^ ions derived from SnCl_2_·2H_2_O to yield tin(II) oxalate (SnC_2_O_4_). The low solubility of SnC_2_O_4_ results in its precipitation as a white gel. Driven by crystal self-assembly, the precipitate forms prismatic structures with uniform morphology. Subsequently, the precursors were calcined in air at temperatures ranging from 300 to 500 °C via a thermal oxidation process. Modulation of the calcination temperature enables the formation of distinct porous architectures. The evolution of internally generated carbon dioxide (CO_2_) during thermal decomposition facilitates the creation of a bulk 3D morphology featuring porous channels, a process governed by a top-down chemical strategy [44,45]. Recent literature on the synthesis of SnO_2_ electrode materials primarily emphasizes hydrothermal, solvothermal and electrochemical deposition methods, among others, which are largely confined to laboratory-scale investigations and yield limited quantities. The scarcity of scalable synthesis routes presents a significant impediment to commercialization. The synthetic pathway presented here for porous SnO_2_ materials offers a promising alternative. More importantly, this synthetic pathway utilizes water as the sole solvent and operates without any surfactants or additional organic solvents. Consequently, the present strategy is cost-effective, environmentally benign, and benefits from readily recoverable water solvents. Further advantages include its scalability and operational simplicity. In a typical synthesis, the yields of the precursor and the final porous SnO_2_ powder were approximately 45.1828 g and 32.2472 g, respectively (see Supporting Information, Figure S1), with potential for further improvement through optimization of reaction parameters (see Supporting Information, Figure S1 and Table S1). Thus, this chemical strategy exhibits distinct advantages over previously reported methods. Its versatility permits extension to other transition metal oxides, enabling the large-scale production of highly porous materials with promising potential for applications in catalysis, electrochemical energy-storage, and sensing.

**Figure 2:**
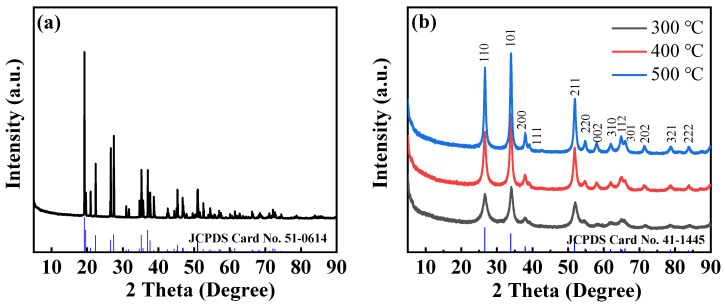
XRD pattern of (**a**) precursor SnC_2_O_4_, and (**b**) SnO_2_ after thermal treatment (300–500 °C).

The crystal structure and phase identification of the as-synthesized precursor were initially characterized by X-ray diffraction (XRD), as shown in Figure 2a. All observed diffraction peaks correspond well to the monoclinic phase of tin oxalate (SnC_2_O_4_, JCPDS Card No. 51-0614). The precursor was subsequently calcined in air at temperatures ranging from 300 to 500 °C for 90 min, resulting in light-yellow powder samples. As shown in Figure 2b, the diffraction peaks observed at 2θ values of 26.6°, 33.8°, 37.9°, 38.9°, 51.7°, 54.7°, 57.8°, 61.8°, 64.7°, 65.9°, 71.2°, 78.7°, and 83.7° correspond to the tetragonal rutile structure (JCPDS Card No. 41-1445) of the SnO_2_ materials. These peaks are indexed to the (110), (101), (200), (111), (211), (220), (002), (310), (112), (301), (202), (321), and (222) lattice planes of SnO_2_ materials, respectively [46]. Apart from SnO_2_ characteristic peaks, no other signals of impurities such as SnC_2_O_4_, SnO and Sn were detected in the prepared samples, indicating complete conversion of the SnC_2_O_4_ precursors into phase-pure SnO_2_. Moreover, the narrow and sharp diffraction peaks of the precursor and the samples annealed at temperatures ranging from 300 to 500 °C suggest high crystallinity. Furthermore, the XRD pattern of sample sintered at 250 °C is also presented in Figure S3 (see Supporting Information). The diffraction profile is predominantly characterized by reflections corresponding to SnC_2_O_4_, along with additional peaks at 2θ values of 33.8°, which are consistent with the presence of SnO_2_.

**Figure 3:**
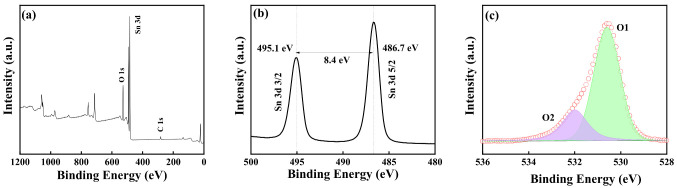
XPS spectra of SnO_2_(300): (**a**) survey spectrum, (**b**) Sn 3d and (**c**) O 1s.

To further investigate the elemental valence and chemical composition of the as-synthesized porous SnO_2_ materials, X-ray photoelectron spectroscopy (XPS) measurements were performed, as presented in Figure 3 and Figures S4 and S5 (see Supporting Information). The XPS survey spectrum of the SnO_2_ sample calcined at 300 °C (Figure 3a) displays prominent peaks assigned to C 1s, O 1s and Sn 3d. The high-resolution Sn 3d spectrum (Figure 3b) exhibits two peaks at binding energies of 486.7 eV (Sn 3d_5/2_) and 495.1 eV (Sn 3d_3/2_), in agreement with previously reported values [47,48]. A spin–orbit splitting energy of 8.4 eV between the two main peaks confirms the presence of Sn in the +4-oxidation state in the SnO_2_(300) sample [49,50]. The peak in the O 1 s XPS spectrum (Figure 3c) could be fitted into two sub-peaks, which demonstrates the broad and asymmetric peaks of the different coordination of oxygen in the nanoparticles. The O1 peak at 530.6 eV was associated with the lattice oxygen (Sn-O bond) in the crystal structure of SnO_2_, and the O_2_ peak at 531.9 eV was related to the insufficient oxygen induced by interstitial oxygen, oxygen vacancy and anti-site oxygen [19,27], demonstrating a high hole quantity inside the oxygen-deficient area. Furthermore, the XPS analysis of both SnO_2_(400) and SnO_2_(500) samples further verifies the complete conversion of tin(II) oxalate to tin(IV) oxide under high-temperature calcination conditions (see Supporting Information, Figures S4 and S5). These XPS results are fully consistent with the XRD data. Figure S6 presents the Raman spectra of the synthesized SnO_2_ materials. The spectra exhibit characteristic peaks at approximately 479.8 cm^−1^ and 618.5 cm^−1^, corresponding to the E_g_ and A_1g_ vibrational modes of the tetragonal rutile SnO_2_ phase, respectively, which are in good agreement with previous reports [6,25]. Additionally, a peak at 243.6 cm^−1^ is observed, which has been attributed to oxygen vacancies by Liu et al. [51]. Specifically, the peaks at 243.6 cm^−1^ and 618.5 cm^−1^ are associated with subbridging and bridging oxygen vacancies, respectively. Notably, the intensities of these oxygen-vacancy-related peaks are significantly higher for the SnO_2_(300) sample compared to those calcined at higher temperatures, indicating a higher concentration of oxygen vacancies in the SnO_2_(300) electrode. These oxygen vacancies enhance the electrochemical performance by serving as active adsorption sites, improving electronic conductivity through donor states near the conduction band, and lowering the energy barrier of the reversible conversion reaction.

**Figure 4:**
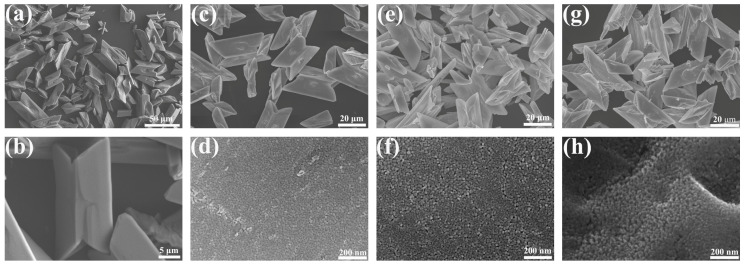
SEM images of the SnC_2_O_4_ precursor (**a**,**b**), SnO_2_(300) (**c**,**d**), SnO_2_(400) (**e**,**f**) and SnO_2_(500) (**g**,**h**).

**Figure 5:**
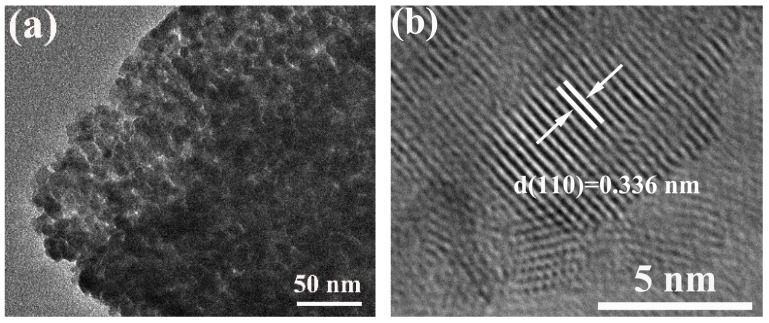
TEM and HRTEM images of the SnO_2_(300) sample (**a**) TEM, (**b**) HRTEM.

The surface morphology of the as-synthesized SnC_2_O_4_ precursor sample was investigated using field-emission scanning electron microscopy (FE-SEM). As depicted in Figure 4a,b, the precursor displayed a uniform, bulk prism-like morphology with a smooth surface. More interestingly, V-shaped openings were consistently observed at both ends of the particles. Following high-temperature calcination, the precursor of SnC_2_O_4_ was converted into SnO_2_, as confirmed by XRD and XPS analyses. The morphological evaluation of the resulting SnO_2_ was also characterized by SEM, as presented in Figure 4c–h. Low-magnification images (Figure 4c,e,g) revealed that the prismatic morphology of the precursor was largely retained after thermal treatment. Higher-magnification SEM images (Figure 4d,f,h) showed that the initially smooth surfaces evolved into a coarse texture composed of numerous nanosized subunits, with diameters ranging from 5 to 20 nm. TEM analysis (Figure 5a) shows that the SnO_2_(300) sample is composed of porous aggregates formed by numerous irregularly shaped nanoparticles (NPs). High-resolution TEM (HRTEM) analysis (Figure 5b) was utilized to elucidate the crystallographic structure of SnO_2_(300) NPs, revealing lattice fringes with an interplanar spacing of 0.336 nm, which corresponds to the *d*-spacing of the (110) planes of tetragonal rutile SnO_2_ [52]. Micro-nano assembled metal oxides, which integrate both micro- and nano-scale structural features, are currently regarded as promising materials for energy-storage applications. The nano-scale components facilitate rapid ion diffusion due to their short ion-transport paths, while also providing a large surface area that increases the number of active sites available for faradaic reactions [53]. Furthermore, micro-scale architecture, distinguished by interparticle pores and interconnected pathways, efficiently mitigates volume changes during ion insertion and extraction processes.

**Figure 6:**
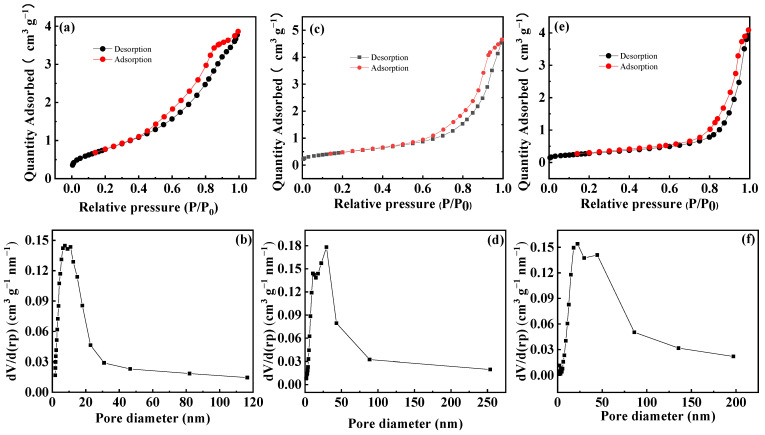
Nitrogen adsorption–desorption isotherms of (**a**) SnO_2_(300), (**c**) SnO_2_(400), (**e**) SnO_2_(500), and the BJH pore-size distribution of (**b**) SnO_2_(300), (**d**) SnO_2_(400), (**f**) SnO_2_(500).

The pore characteristics between adjacent particles were clearly visible from the SEM and TEM test. Such unique 3D spatial architecture with porous channels among these nanoparticles can provide a high specific surface area (SSA), which facilitates electrolyte infiltration and rapid ions diffusion, thereby enhancing the electrochemical energy-storage performance. The porous structures of these SnO_2_ samples are analyzed by using the N_2_ adsorption–desorption isothermal measurements, as presented in Figure 6. The SSA was calculated to be 65.99 m^2^ g^−1^, 39.79 m^2^ g^−1^ and 23.26 m^2^ g^−1^ for SnO_2_(300), SnO_2_(400) and SnO_2_(500), respectively. Electrode materials with larger SSA can provide more active sites for the occurrence of redox reactions, thereby facilitating more efficient utilization of electrode materials. As depicted in Figure 6a, the N_2_ adsorption–desorption behavior of the SnO_2_(300) sample exhibits a type IV curve with an H2 hysteresis loop. In comparison, both SnO_2_(400) and SnO_2_(500) possess the isotherms of type-IV feature and type-H3 hysteresis loop, indicating the presence of mesoporous structure in the SnO_2_(300) sample [19]. The pore-size distribution (PSD) of as-prepared SnO_2_ samples was further determined by the Barrett–Joyner–Halenda (BJH) method (Figure 6b,d,f), revealing the PSD of 8.97, 22.45 and 27.39 nm for the SnO_2_(300), SnO_2_(400) and SnO_2_(500), respectively. Notably, the SnO_2_(300) exhibits a smaller average pore size than the other materials, which corresponds to its increased surface area. These optimized pores can provide effective channels for ion transportation and shorten the distance for electrolyte diffusion to induce more absorption of ions and facilitate the rapid faradaic process, which is beneficial for improving electrochemical performance [54,55]. Notably, this bulk architecture assembled from nanoparticles not only alleviates the aggregation of active materials during the electrochemical reaction but also retains the advantages of nanomaterials, such as a high specific surface area, to enhance electrochemical performance.

Based on the aforementioned results, such 3D porous SnO_2_ architectures, characterized by their high surface area, abundant active sites, and optimized porosity structures, promote efficient ion and electron transport during electrochemical reactions, rendering them promising electrode candidates for supercapacitors. The electrochemical energy-storage properties and behavior of these porous SnO_2_-based materials calcined at various temperatures, were systematically evaluated using various electrochemical techniques, including cyclic voltammetry (CV), galvanostatic charge/discharge (GCD), and electrochemical kinetics analysis.

**Figure 7:**
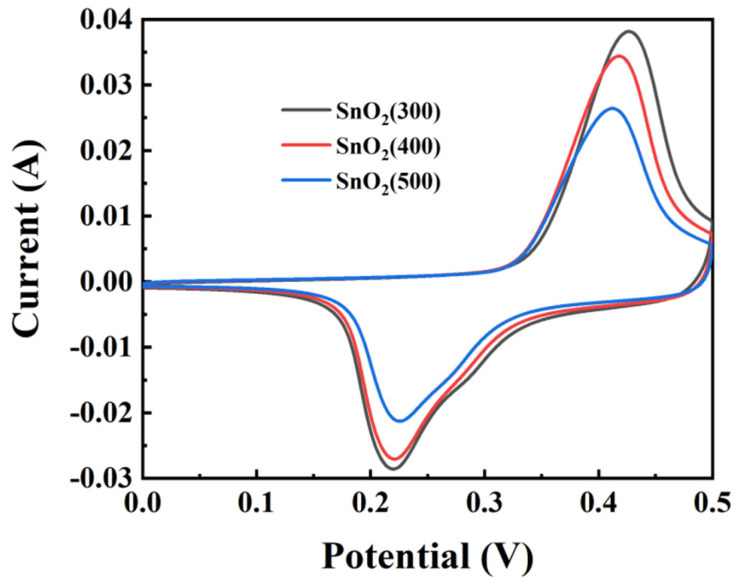
CV curves of porous SnO_2_ samples calcined at different temperatures (300–500 °C) at a scan rate of 50 mV s^−1^.

To evaluate the electrochemical performance of the as-prepared porous SnO_2_-based samples, CV measurements were initially performed in a standard three-electrode system at a fixed scan rate of 50 mV s^−1^ using a KOH (2 mol L^−1^) aqueous electrolyte. As illustrated in Figure 7, the CV curve of the SnO_2_(300) electrode exhibits a greater enclosed area and higher current responses compared to those of SnO_2_(400) and SnO_2_(500) electrodes, measured within a potential window of 0.0 to 0.5 V at identical scan rates. The integrated CV area of SnO_2_(300) is 1.1 and 1.3 times greater than that of SnO_2_(400) and SnO_2_(500), respectively, indicating its superior specific capacitance, as the enclosed CV area is proportional to capacitance [56,57]. Furthermore, the presence of well-defined redox peaks in both cathodic and anodic regions suggest pseudo-capacitive behavior, confirming the battery-like characteristics of the SnO_2_-based electrode materials [14]. The corresponding faradaic reactions can be expressed as follows [16,19,33,58]:

(1) $${\mathrm{SnO}}_{2}\;+\;H_{2}O\;+\;e^{-}\;↔\;\mathrm{SnOOH}\;+\;{\mathrm{OH}}^{-}$$

(2) $$\mathrm{SnOOH}\;+\;e^{-}\;↔\;\mathrm{SnO}\;+\;{\mathrm{OH}}^{-}$$

**Figure 8:**
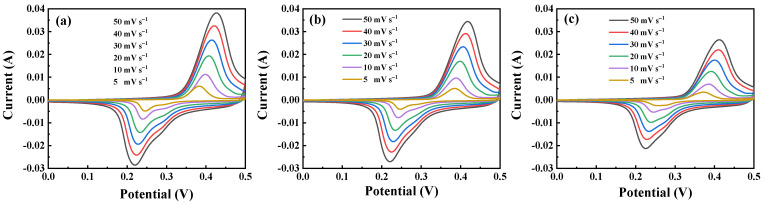
CV curves of (**a**) SnO_2_(300), (**b**) SnO_2_(400) and (**c**) SnO_2_(500) electrodes at different scan rates: from 5 to 50 mV s^−1^.

Therefore, according to Equations (1) and (2), this pair of peaks (0.22/0.41 V vs. Hg/HgO) could be attributed to the redox reactions SnO_2_/SnO [16,19,33,58]. Furthermore, the CV test of SnO_2_-based electrodes was investigated in the potential range of 0.0–0.5 V at diverse applied potential scan rates, as the results were shown in Figure 8. The peaks of the CV curve widen continuously with the scan rate boosting from 5 to 50 mV s^−1^, and the enclosed area under the CV loops expands progressively while the peak type of the CV curve almost remains the same, indicating the good reversibility and rate capability of the present SnO_2_-based porous electrode materials. The faradaic reactions of SnO_2_-based electrodes are the source of redox peaks, which are clearly seen in all the CV curves. When the scan rate increases in CV tests, the apparent shifts in the anodic (oxidation) peak toward more positive potentials and the cathodic (reduction) peak toward more negative potentials are observed, which can be attributed to several interrelated electrochemical and physical phenomena, such as ohmic resistance, kinetic polarization and mass transport effects [59,60,61,62]. To evaluate the possible contribution of the acetylene black (AB) additive to the measured capacitance, a control electrode was fabricated using only AB and PVDF binder with the same mass ratio and tested under identical three-electrode conditions. As shown in Figure S7 (see Supporting Information), the CV curve of pristine AB exhibits a quasi-rectangular shape characteristic of carbon-based electrical double-layer capacitance (see inset of Figure S7). However, the integrated area of the AB curve is negligible compared to that of the SnO_2_(300) electrode. This result confirms that the capacitance contribution from the AB additive is minimal, and the observed electrochemical performance is dominated by the porous SnO_2_(300) material.

**Figure 9:**
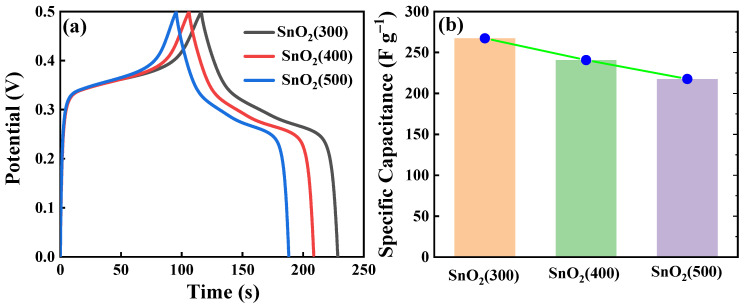
(**a**) GCD curves of SnO_2_-based electrode at a current density of 1 A g^−1^; (**b**) the specific capacitance of SnO_2_-based electrode at a current density of 1 A g^−1^.

To further evaluate the electrochemical performance, galvanostatic charge/discharge (GCD) measurements were carried out on the as-synthesized porous SnO_2_-based electrodes within a potential window of 0.0 to 0.5 V. A detailed comparison of the GCD profiles for these electrodes at a current density of 1 A g^−1^ is presented in Figure 9. As shown in Figure 9a, all curves exhibit a non-linear triangular shape with distinct redox plateaus, suggesting pseudo-capacitive characteristics of the porous SnO_2_ materials [16,19,33,58], consistent with the conclusions derived from CV analysis. Notably, the SnO_2_(300) electrode exhibits a prolonged discharge duration relative to the SnO_2_(400) and SnO_2_(500) samples at a current density of 1 A g^−1^, indicative of a higher specific capacitance. These findings demonstrate that the SnO_2_(300) electrode possesses excellent electrochemical performance. The discharge specific capacitance of the porous SnO_2_-based electrodes was determined using Equation (7) (Section 4.3). As shown in Figure 9b, the SnO_2_(300) electrode exhibits a higher specific capacitance than the SnO_2_(400) and SnO_2_(500) electrodes.

**Figure 10:**
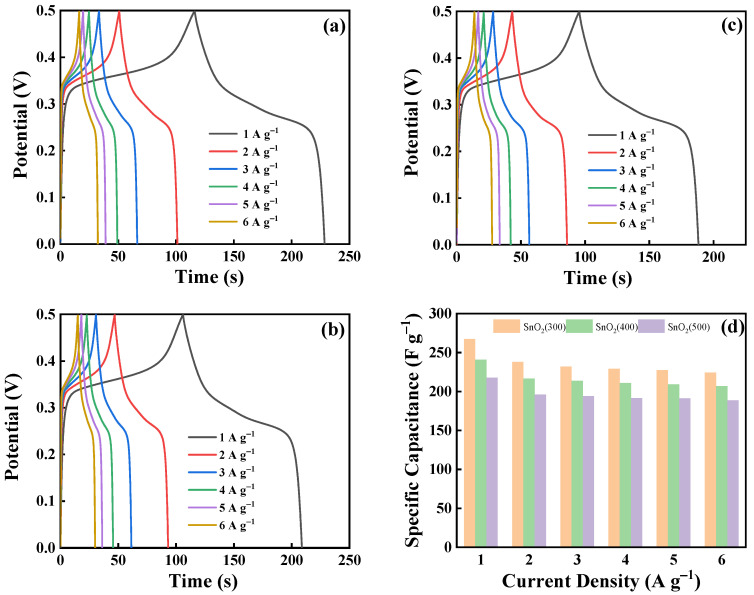
GCD curves of SnO_2_ electrode at different current densities from 1 to 6 A g^−1^: (**a**) SnO_2_(300); (**b**) SnO_2_(400); (**c**) SnO_2_(500); (**d**) rate capability.

To gain deeper insight into the charge-storage behavior, GCD measurements were performed at current densities ranging from 1 to 6 A g^−1^ within a potential window of 0.0 to 0.5 V (Figure 10). The GCD profiles of all porous SnO_2_ electrodes deviate from ideal triangular shapes, indicating a pseudo-capacitive charge-storage mechanism dominated by fast faradaic processes (Figure 10a–c). The corresponding capacitance values, calculated from Equation (7), are summarized in Figure 10d. Furthermore, the SnO_2_(300) electrode delivers a specific capacitance of 267.31 F g^−1^ (133.66 C g^−1^) at 1 A g^−1^, substantially higher than those of SnO_2_(400) (240.76 F g^−1^, 120.38 C g^−1^) and SnO_2_(500) (217.62 F g^−1^, 108.81 C g^−1^) under the same current conditions. Furthermore, at current densities of 1, 2, 3, 4, 5 and 6 A g^−1^, the capacitances of SnO_2_(300) are 267.31, 237.88, 232.07, 229.12, 227.54 and 224.13 F g^−1^, respectively; for SnO_2_(400), the values are 240.76, 216.56, 213.62, 210.88, 209.07 and 206.89 F g^−1^; and for SnO_2_(500), they are 217.62, 196.15, 193.94, 191.46, 191.08 and 188.59 F g^−1^.

**Figure 11:**
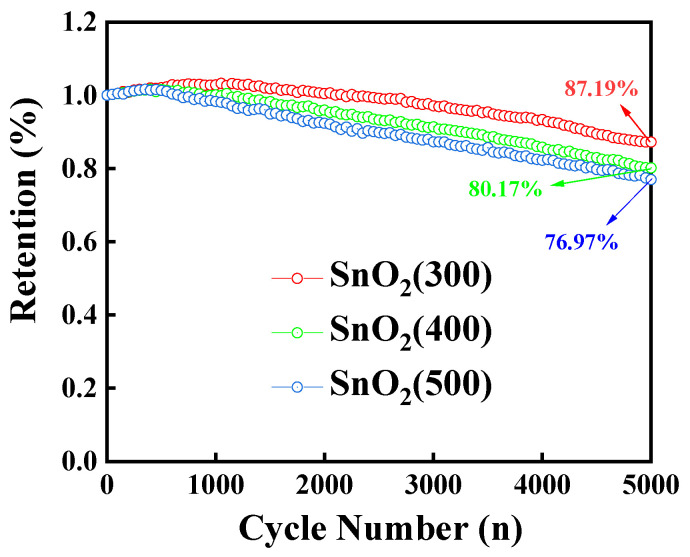
Cycle stability performance of SnO_2_ electrode at current density of 1 A g^−1^.

The gradual decline in capacitance at higher scan rates is mainly due to the limited diffusion time of electrolyte ions, which restricts the utilization of deeper active sites for faradaic reactions [57]. As observed, the GCD profile of the porous SnO_2_ electrode exhibits only minor variations between current densities of 1 and 6 A g^−1^, reflecting stable charge-storage characteristics and improved kinetics of faradaic processes. The long-term cycling stability, an important parameter for supercapacitor applications, was evaluated with continuous GCD tests over 5000 cycles at a current density of 1 A g^−1^, as illustrated in Figure 11. Initially, the specific capacitance of the SnO_2_(300) electrode slightly increases during the first ~1000 cycles, attributable to an electrochemical activation process in the porous structure, and subsequently stabilizes, maintaining nearly constant performance throughout the remainder of the cycling test. This behavior indicates effective stabilization of the electrode–electrolyte interface and robust mechanical integrity of the material under prolonged cycling. After 5000 cycles, the SnO_2_(300) electrode demonstrates satisfactory cycling stability, retaining 87.19% of its initial capacitance. The SnO_2_(400) and SnO_2_(500) samples also exhibit satisfactory performance under the same current density, achieving efficiencies of 80.17% and 76.97%, respectively. Notably, the as-prepared porous SnO_2_ electrodes exhibit pronounced battery-type behavior [9,18,19,43]. Consequently, the capacitance loss after 5000 cycles is primarily attributed to mechanical pulverization caused by repeated volume changes in SnO_2_ during subsequent reversible insertion/extraction, which partially disrupts the conductive network [27,34]. However, compared to the samples calcined at 400 °C and 500 °C, the SnO_2_(300) sample demonstrates superior electrochemical performance. This observation suggests that although electrodes with battery-type characteristics inevitably undergo degradation due to volume expansion, optimizing the calcination temperature to tune the pore structure and increase the specific surface area can effectively enhance the energy-storage performance of the material [9,18,19,43]. The uniform micro/nano-assembled architecture of SnO_2_, characterized by porous features and nano-scale pathways, enables efficient infiltration of ions into the bulk of the active material and enhances the electrochemical redox reactivity across the entire electrode [16,19,23].

These findings revealed the enhanced electrochemical energy-storage performance of porous SnO_2_-based electrodes in supercapacitors. To gain further insight into the charge-storage mechanisms and electrochemical kinetics of these porous SnO_2_-based materials, the power law relationship between the peak current (*i_p_*) and scan rate (*v*) in the CV profiles was analyzed, as expressed by the following equations [63]:

*i_p_* = *a*
*v^b^* (3)

log *i_p_* = *b* log *v +* log *a* (4)

**Figure 12:**
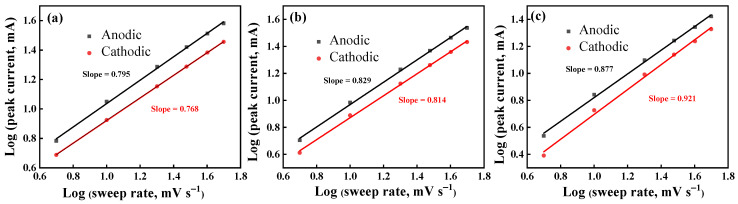
The relationship between anodic/cathodic peak current and scan rate: (**a**) SnO_2_(300), (**b**) SnO_2_(400) and (**c**) SnO_2_(500).

Within this equation, the predictable *b*-value serves as a key parameter for assessing energy-storage mechanisms. Theoretically, a *b*-value of 0.5 indicates an ion diffusion-controlled process, whereas a value of 1.0 corresponds to a surface-controlled capacitive process [64]. As derived from the linear regressions of log *v* versus log *i_p_* plots (Figure 12a–c), the calculated *b*-values for the SnO_2_(300) electrode are 0.795 and 0.768; for SnO_2_(400), 0.829 and 0.814; and for SnO_2_(500), 0.877 and 0.921. Thus, these results indicate that charge storage in SnO_2_-based electrodes exhibits a synergistic pseudo-capacitive behavior, involving both surface-controlled capacitive and ion diffusion processes [63,64]. The underlying reason is that as the temperature increases, SnO_2_ particles undergo Ostwald ripening, leading to larger crystallite sizes and reduced specific surface area. Consequently, electrolyte infiltration becomes less efficient, limiting ion diffusion into the bulk material and shifting the charge-storage mechanism toward more surface-controlled capacitive behavior.

To quantitatively evaluate the specific contributions of surface-controlled and ion diffusion-controlled processes in the as-fabricated porous SnO_2_-based electrodes, Dunn’s method was applied using the following equation [63,64]:

*i*(*v*) = *k*_1_*v* + *k*_2_*v*^1/2^ (5)

*i_v_*/*v*^1/2^ = *k*_1_*v*^1/2^ + *k*_2_ (6)

**Figure 13:**
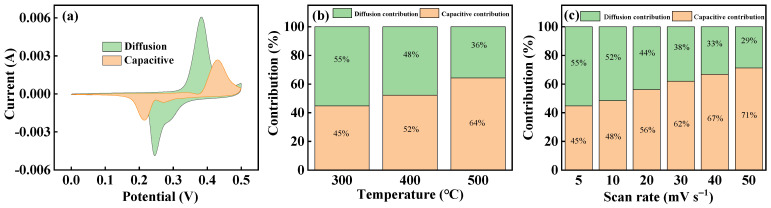
(**a**) The CV curves of capacitive and diffusion contribution at 5 mV s^−1^; (**b**) percentage of capacitive and diffusion contribution at 5 mV s^−1^ for SnO_2_(300), SnO_2_(400) and SnO_2_(500) samples; (**c**) percentage of capacitive and diffusion contribution at various scan rates for SnO_2_(300) samples.

In the equation, the terms *k*_1_*v* and *k*_2_*v*^1/2^ correspond to current contributions from a surface-controlled capacitive and ion diffusion-controlled process, respectively. By analyzing CV profiles at various scan rates (5–50 mV s^−1^), the values of *k*_1_ and *k*_2_ can be obtained from the slope and intercept of the current versus *v*^1/2^ plot [65,66]. At a scan rate of 5 mV s^−1^, for example, the capacitive contribution of SnO_2_(300) was determined to be 45%, while the corresponding ion diffusion contribution comprised the remaining 55% (Figure 13a). At a lower scan rate, the ions in KOH electrolyte can easily enter the SnO_2_ electrodes, thus fully participating in the Faradaic process. As shown in Figure 13b, the contribution from an ion diffusion-controlled process decreased as the sintering temperature increased under a constant scan rate. When the temperature was raised to 400 and 500 °C, the surface capacitive contribution increased to 52% and 64%, respectively.

The quantitative analysis on the contributions of the surface-controlled and ion diffusion-controlled processes of SnO_2_(300) electrode materials at various scan rates is summarized, with the corresponding percentages illustrated in Figure 13c. According to the calculations, when the scan rate rises, the capacitive contribution of SnO_2_(300) electrode increases progressively, reaching 71% at a scan rate of 50 mV s^−1^. At lower scan rates, the electrochemical response is dominated by ion diffusion-controlled processes, since the ions possess adequate time to access the greater part of electrode surface. However, as the scan rate increases, charge storage becomes progressively governed by surface-controlled capacitive processes, which are driven by rapid electron transfer and the availability of surface-accessible active sites [67]. This transition highlights the material’s adaptability across varying charge–discharge conditions, rendering it particularly well suited for hybrid energy-storage systems that require both high energy and power densities.

**Figure 14:**
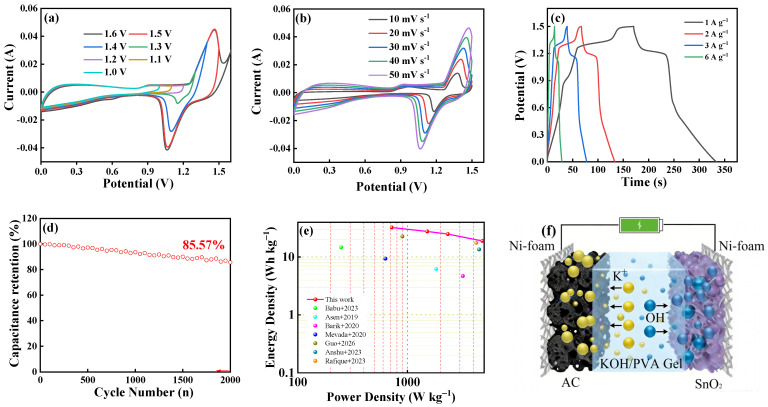
(**a**) CV curves of the P-SnO_2_(300)//AC asymmetric supercapacitors for the different voltage window from 1.0 to 1.6 V at 40 mV s^−1^; (**b**) CV curves at different scan rates (10–50 mV s^−1^) of P-SnO_2_(300)//AC; (**c**) GCD profiles of P-SnO_2_(300)//AC under a current load of 1, 2, 3 and 6 A g^−1^; (**d**) cycle stability performance of P-SnO_2_(300)//AC at current density of 1 A g^−1^; (**e**) Ragone plots of SnO_2_(300) and some other SnO_2_-based supercapacitors reported previously; (**f**) a schematic illustration of the P-SnO_2_//AC device using the PVA/KOH gel as electrolyte [33,74,75,76,77,78,79].

Electrochemical analysis with a three-electrode system revealed that the SnO_2_(300) samples possess superior energy-storage performance compared to other SnO_2_ samples (SnO_2_(400) and SnO_2_(500)). To evaluate the practical applicability of such porous SnO_2_ materials, an asymmetric supercapacitor (ASC) device denoted as P-SnO_2_//AC was constructed. The device employed SnO_2_(300) as the positive electrode and activated carbon (AC) as the negative electrode, with a PVA/KOH gel electrolyte, to extend the operational voltage window [68,69,70]. The AC materials can serve as a suitable anode in P-SnO_2_//AC supercapacitor due to their large-scale production and chemical stability [71,72]. To optimize the electrochemical performance of the P–SnO_2_//AC supercapacitor, the mass ratio of the positive to negative electrode was carefully tuned to achieve a balanced charge distribution according to Equation (8) (Section 4.3). Figure S8 presents the CV profiles of porous SnO_2_(300) cathode and AC anode electrode, measured at a scan rate of 50 mV s^−1^ using a three-electrode configuration. The AC electrode exhibits a nearly rectangular profile, characteristic of electric double-layer capacitive behavior within the potential window of −1.0–0.0 V, while the porous SnO_2_(300) electrode displays pronounced redox peaks in the potential window of 0.0–0.5 V, indicative of faradaic pseudo-capacitance. To ensure the suitable voltage window of the hybrid supercapacitor device, CV measurements of the assembled ASC (P–SnO_2_//AC) were performed over potential windows ranging from 0.0 to 1.6 V at a scan rate of 40 mV s^−1^, as illustrated in Figure 14a. When the applied voltage was increased from 1.0 to 1.6 V, a distinct polarization phenomenon was observed in the CV curve. It is evident that the stable potential window can be extended up to 1.5 V, which is also consistent with the sum of the potential windows of AC (−1.0–0.0 V) and SnO_2_(300) (0.0–0.5 V). Therefore, a voltage window of 0.0–1.5 V was selected for all subsequent electrochemical measurements in the two-electrode configuration. From the CV analysis it can be observed that all the curves have retained similar shapes even after raising the cell voltage up to 1.5 V. Figure 14b,c depict the CV and GCD profiles of the P-SnO_2_//AC device at various scan rates and current densities in 1.5 V. The well-maintained CV profiles at progressively increasing scan rates indicate excellent electrochemical reversibility of the assembled P-SnO_2_//AC supercapacitor [73]. Based on the GCD curves, the specific capacitance of the ASC was calculated using Equation (7). The P-SnO_2_//AC device exhibited a specific capacitance of 103.96 F·g^−1^ at a current density of 1 A·g^−1^. Even at a high current density of 6 A g^−1^, a specific capacitance of 60.80 F g^−1^ can still be attained. Beyond specific capacity and rate performance, cycling durability is another key criterion for assessing the commercial feasibility of P-SnO_2_//AC. Figure 14d presents the capacitance retention of P-SnO_2_//AC as a function of cycle number over 2000 GCD cycles, measured at a current density of 1 A g^−1^. As a result, the device retains 85.57% of its initial capacitance after 2000 cycles, demonstrating excellent cycling stability. The energy density (*D_e_*) and power density (*D_p_*) of the ASC are determined using Equations (9) and (10), respectively. The fabricated device exhibited a *D_e_* of 32.49 Wh kg^−1^ along with a high *D_p_* of 718.97 W kg^−1^. Furthermore, it maintained a *D_e_* of 19 Wh kg^−1^ while achieving a remarkable *D_p_* of 4816.56 W kg^−1^. Figure 14e showed the Ragone plots regarding the P-SnO_2_//AC devices assembled with SnO_2_-based electrodes [33,74,75,76,77,78,79]. Based on the above analysis, the as-synthesized porous SnO_2_ electrode materials exhibit superior electrochemical performance and show promise as an advanced electrode material for high-performance supercapacitor devices. To evaluate the practical energy-storage capability of the as-prepared P-SnO_2_, a solid-state asymmetric supercapacitor was assembled with AC as the counter electrode and PVA/KOH gel as the quasi-solid-state electrolyte, as schematically shown in Figure 14f. In this two-electrode configuration, the P-SnO_2_ and AC electrodes were directly coated onto nickel foam current collectors, with the PVA/KOH gel serving as both the separator and the ionic conductor. The operating voltage window of the P-SnO_2_//AC device could be extended to 1.5 V, which is significantly higher than that of a symmetric P-SnO_2_ device (typically limited to ~0.5 V in an aqueous electrolyte). This voltage extension is attributed to the asymmetric design, where the positive potential is largely sustained by the P-SnO_2_ electrode and the negative potential by the AC electrode, effectively preventing hydrogen evolution at the negative side. The use of a gel electrolyte also enables the fabrication of thin, flexible, and safe energy-storage devices suitable for next-generation wearable electronics.

## 3. Conclusions

In summary, a bulk 3D morphology SnO_2_ electrode material with porous channels was synthesized via a facile thermal oxidation gas-release route. The present synthetic method is straightforward, requires no surfactant, and is readily scalable, in accordance with the principle of green chemistry. Benefiting from the porous architecture with larger specific surface area and more active sites, the as-synthesized SnO_2_-based materials allow electrolyte ions to enter more easily the interior of the bulk materials through the porous channel, thereby improving the reaction kinetics and charge-storage performance. The results show that the as-synthesized porous SnO_2_-based electrode materials with pseudo-capacitive behavior exhibit excellent electrochemical energy-storage performance, delivering a high specific capacity of 267.31 F g^−1^ at 1 A g^−1^ in a symmetric capacitor design. Furthermore, the SnO_2_(300) retains 87.19% of its initial capacitance after 5000 cycles under 1 A g^−1^. Moreover, when assembled into an asymmetric supercapacitor device with active carbon as the negative electrode, SnO_2_(300)//AC device achieves energy density of 32.49 Wh kg^−1^ at power density of 718.97 W kg^−1^ in the potential window of 0–1.5 V. Kinetic analysis revealed a mixed charge-storage mechanism involving both diffusion-controlled faradaic processes and surface-controlled capacitive contributions. The present work provides an effective and reliable strategy for the large-scale preparation of porous metal oxide materials, contributing to the advancement of electrochemical energy storage.

## 4. Materials and Methods

### 4.1. Sample Synthesis

All raw materials utilized in this investigation were of analytical reagent (A.R.), and the deionized water was used throughout the experimental procedures. To synthesize porous SnO_2_ materials for application as electrodes in high-performance supercapacitors, 55.6250 g of stannous chloride dehydrate (SnCl_2_·2H_2_O, ≥98.0%, Sinopharm Chemical Reagent Co., Ltd., Shanghai, China) was dispersed in 2500 mL of distilled water. The mixture was magnetically stirred at room temperature for 9 min, resulting in a homogeneous milky-white solution (designated as solution A). Meanwhile, a solution of oxalic acid dihydrate (H_2_C_2_O_4_·2H_2_O, ≥99.8%, Aladdin Chemical Reagent Co., Ltd., Shanghai, China) was prepared according to the stoichiometric ratio (1:1) of the precursor under identical stirring conditions, yielding a clear solution (designated as solution B). Subsequently, the precursor of the porous SnO_2_ material was obtained by gradually adding solution B into solution A under continuous stirring. The reaction mixture was covered and maintained at 35 °C in a water bath for 15 min. After standing at room temperature for 6 h, the white wet gel formed at the bottom of the reaction beaker was collected by centrifugation, washed repeatedly with distilled water, and then vacuum dried at 75 °C for 6 h. The as-synthesized precursor was then transferred into a muffle furnace and calcined at temperatures ranging from 300 to 500 °C for 1.5 h with a heating rate of 5 °C min^−1^, resulting in a light-yellow powder. The resulting samples were labeled as SnO_2_(300), SnO_2_(400) and SnO_2_(500), respectively. Finally, the calcined products were thoroughly ground and stored in an airtight container at room temperature for subsequent use.

### 4.2. Materials Characterization

The crystalline phase and structure of the as-fabricated materials were characterized by X-ray powder diffractometry (XRD, Rigaku 9 kW, Tokyo, Japan) with a 2θ range of 5° to 90° at a scan rate of 20° min^−1^. The surface morphology and microstructure features of the prepared samples were examined using a field-emission scanning electron microscopy (FE-SEM, S-4800, Hitachi, Tokyo, Japan) and transmission electron microscopy (TEM, JEOL JEM-F200, Tokyo, Japan). An X-ray photoelectron spectrometer (XPS, AXIS SUPRA, Manchester, UK) was employed to analyze the surface element composition and the valence states of Sn and O in the SnO_2_ powder. The specific surface areas and pore-size distributions of samples were determined from nitrogen adsorption–desorption isotherm measured on an ASAP 2020 analyzer (Micromeritics, Norcross, GA, USA). Prior to conducting N_2_ adsorption–desorption analyses, all samples were degassed at 160 °C for 12 h.

### 4.3. Electrochemical Measurements

The electrochemical performance of the porous SnO_2_ electrode materials was evaluated using a CHI-660E electrochemical workstation (Shanghai Chenhua Co. Ltd., Shanghai, China). Firstly, the working electrode was prepared by blending the porous SnO_2_ powder, acetylene black (AB), and polyvinylidene fluoride (PVDF) in a mass ratio of 80:10:10. Then, an appropriate amount of N-methyl-2-pyrrolidone (NMP) solvent was added, and the mixture was ground thoroughly for 15 min to yield a homogeneous black slurry in an agate mortar. After that, the resulting slurry was uniformly coated onto a pretreated nickel foam current collector (1 × 1.2 cm^2^) using the brush coating method. The coated nickel foam was then dried at 75 °C for 7 h in a vacuum oven. Finally, the electrodes were pressed under a pressure of 5 MPa for 50 s. The active material mass loading is 0.8–1.2 mg. In a conventional three-electrode configuration, the as-prepared porous SnO_2_ electrode served as the working electrode, with a platinum plate and a Hg/HgO electrode employed as the counter and reference electrodes, respectively. The electrochemical measurements, including cyclic voltammetry (CV), galvanostatic charge–discharge (GCD), and electrochemical kinetics analysis, were carried out in a 2 M KOH aqueous electrolyte.

The specific capacitance *C_s_* of the porous SnO_2_ electrodes was calculated based on the GCD curves using the following equation [33,80,81]:

(7) $$C_{s}=\frac{2i_{m}\int Vdt}{V^{2}{|}_{v_{i}}^{v_{f}}}$$

where *i_m_* (A g^−1^) represents the current density of the working electrode, *∫Vdt* refers to the integrated current area under the discharge portion of the GCD curve, and *v_i_* and *v_f_* are the initial and final potentials of the discharge window, respectively.

To evaluate the practical application of the porous SnO_2_ materials, an asymmetric supercapacitor (ASC) was fabricated using them as the positive electrode and activated carbon (AC, Nanjing XFNANO Materials Tech Co., Ltd., Nanjing, China) as the negative electrode. The KOH/PVA gel (PVA, Polyvinyl alcohol, Sinopharm Chemical Reagent Co., Ltd., Shanghai, China) was employed as electrolyte [82]. 2.0 g of PVA and 2.24 g KOH were individually added into 20 mL of deionized water. All the solutions were mixed under stirring at 85 °C until the solution became a transparent gel and finally cooled down to room temperature. Prior to this, according to the charge balance theory, the optimal mass ratio between the porous SnO_2_ and AC electrodes was determined by the following equation [83]:

(8) $$\frac{m_{+}}{m_{-}}=\frac{C_{s}^{-}\times △V^{-}}{C_{+}\times △V_{+}}$$

where *m*_+_ (g) and *m*_−_ (g) are the mass loading of the porous SnO_2_ (positive) and AC (negative) electrodes, respectively. *C*^+^ (F g^−1^) and *C*^−^ (F g^−1^) represent their specific capacitance of porous SnO_2_ and AC electrodes, respectively. *V*^+^ and *V*^−^ denote the potential window of positive and negative electrodes measured in the three-electrode system, respectively. In addition, the energy density (*D_e_*) and power density (*D_p_*) of ASC device were calculated using the following equations [84,85]:

(9) $$E=\frac{C_{s\times }∆V^{2}}{7.2}$$

(10) $$P=\frac{3600\times E}{∆t}$$

where *C_s_* (F g^−1^) is the specific capacitance of the device, ∆*V* (V) is the operating voltage window, and Δ*t* (s) is the discharge time.

## Data Availability

Data are contained within the article and Supplementary Materials.

## Supplementary Materials

The following supporting information can be downloaded at: https://www.mdpi.com/article/10.3390/gels12060476/s1, Figure S1: Photograph of the precursors and final products synthesized with different amounts of SnCl_2_×2H_2_O. Figure S2: XRD pattern of (a) precursor SnC_2_O_4_, (b) SnO_2_(300), (c) SEM images of the SnC_2_O_4_ precursor, (d) SnO_2_(300) obtained from SnCl_2_·2H_2_O masses of 111.2500 g. Figure S3: XRD pattern of SnO_2_ sample sintered at 250 °C, Figure S4: XPS spectra of SnO_2_(400), Figure S5: XPS spectra of SnO_2_(500), Figure S6: Raman spectra of SnO_2_ samples, Figure S7: The CV curves of P-SnO_2_(300) and AB at a scan rate of 50 mV s^−1^ in the three-electrode system.(Inset: CV of AB alone under identical conditions), Figure S8: The CV curves of P-SnO_2_(300)//AC measured at a scan rate of 50 mV s^−1^ in the three-electrode system, Table S1. The synthetic methods, morphology, specific capacity (SC, 1 A g^−1^), Sn source, theoretical yield (TY, 100% conversion to SnO_2_), and actual yield (AY) of some SnO_2_-based electrode materials [6,32,33,85,86,87,88,89,90,91].
