# Supplementary material for: Thermal Oxidation Gas-Release Strategy for Scalable Synthesis of Porous SnO2 Towards High-Performance Supercapacitor
Source: Gels. 2026 May 29;12(6):476. doi: 10.3390/gels12060476 (PMC13298597; doi:10.3390/gels12060476)
Supplement: Supplementary file 1 [file gels-12-00476-s001.zip › gels-4279724-supplementary.pdf]

Supplementary Material

Thermal Oxidation Gas-Release Strategy for Scalable Synthesis of Porous SnO2 Towards High-Performance Supercapacitor

Xiaoli Wang [1] and Xinyu Zhao [2,3,*]

[1] Liaoning Provincial Key Laboratory of Energy Storage and Utilization, College of Chemistry and Environment Engineering, Yingkou Institute of Technology, Yingkou 115014, China; wangxl@yku.edu.cn
[2] College of Chemistry and Materials Science, Inner Mongolia Minzu University, Tongliao 028000, China
[3] National Demonstration Center for Experimental Chemical Education, Inner Mongolia Minzu University, Tongliao 028000, China
* Correspondence: xyzhao@imun.edu.cn

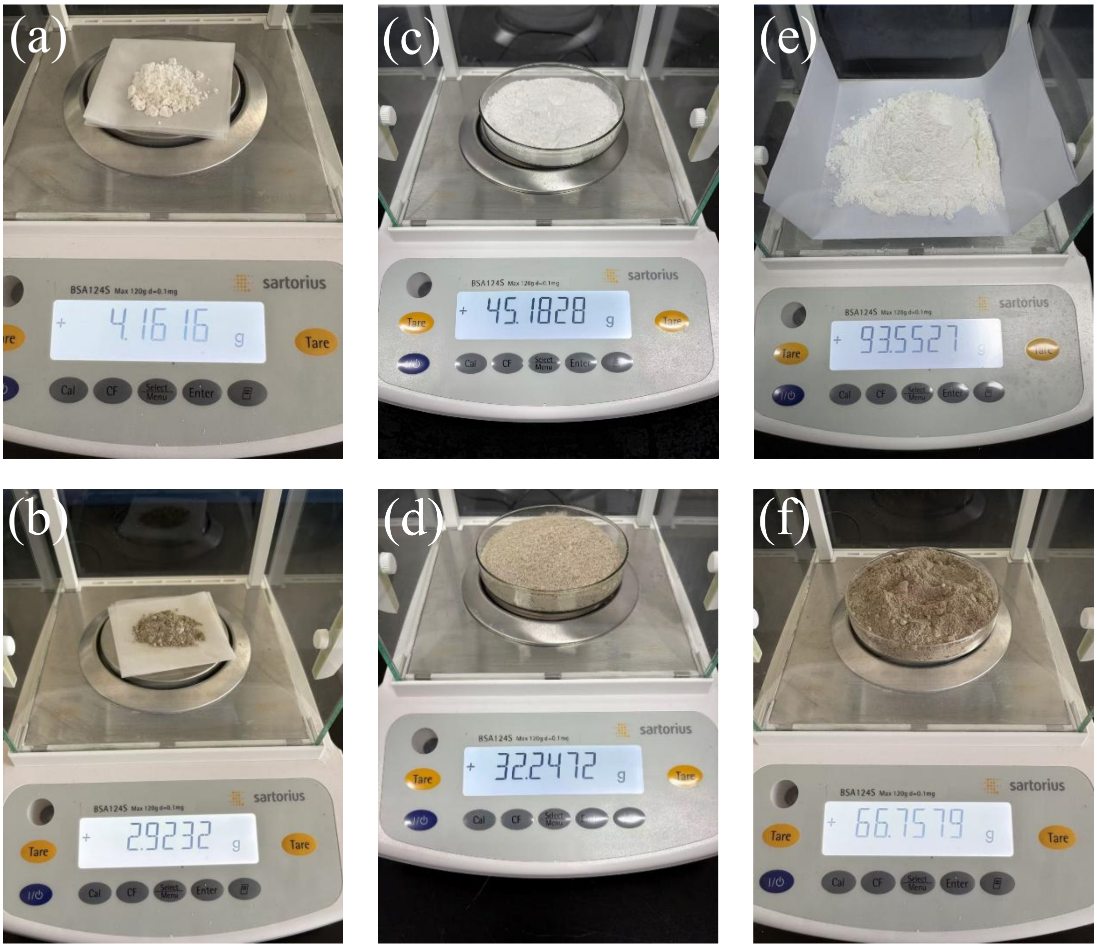

Figure S1. Photograph of the precursors and final products synthesized with different amounts of $SnCl_2 \cdot 2H_2O$. (a, c, e) As-prepared SnC_2O_4 precursors obtained from $SnCl_2 \cdot 2H_2O$ masses of 5.5625 g, 55.6250 g, and 111.2500 g, respectively; (b, d, f) Corresponding SnO_2 samples after thermal treatment at 300 °C.

To evaluate the scalability of the thermal oxidation gas-release route, we performed scale-up experiments by varying the amount of $SnCl_2{\cdot}2H_2O$ (5.5625 g, 55.6250 g, and 111.2500 g), yielding 2.9232 g, 32.2472 g, and 66.7579 g of porous SnO_2 per batch, with corresponding yields of 78.8%, 86.8%, and 89.8%, respectively. The progressive increase in yield is attributed to the fact that the absolute mass loss during washing, drying, and calcination remains relatively constant across batches; therefore, when the starting amount is small, this loss accounts for a larger proportion, resulting in a lower yield. XRD and SEM characterization (Figure S2) confirmed that the crystal structure and porous morphology of all samples were nearly identical to those of the standard batch, indicating excellent process robustness. Although a yield of 66.7579 g per batch represents a laboratory-scale demonstration, the process is readily transferable to industrial settings. Specifically, the gel precursor forms spontaneously in water without the need for organic solvents, surfactants, or corrosive etchants, eliminating concerns regarding reactor corrosion. Furthermore, the calcination step requires only air, and the absence of template removal and subsequent washing steps significantly reduces waste generation.

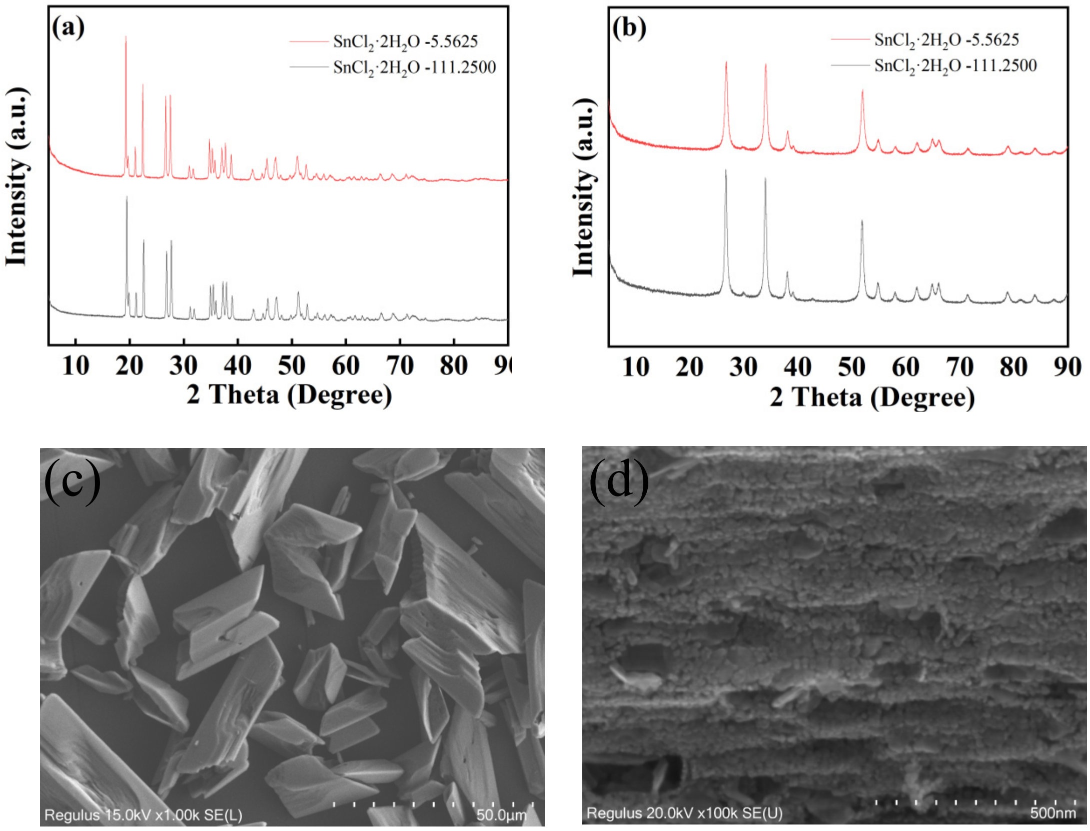

Figure S2. XRD pattern of (a) precursor SnC_2O_4, (b) SnO_2(300), (c) SEM images of the SnC_2O_4 precursor, (d) SnO_2(300) obtained from $SnCl_2{\cdot}2H_2O$ masses of 111.2500 g.

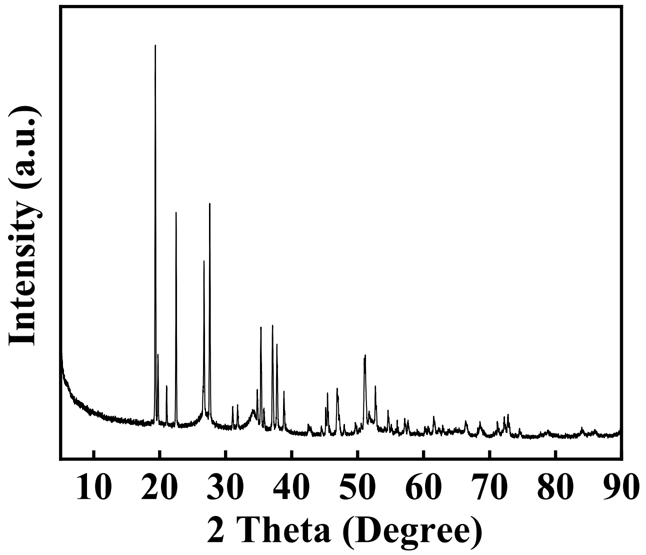

Figure S3. XRD pattern of SnO_2 sample sintered at 250 °C.

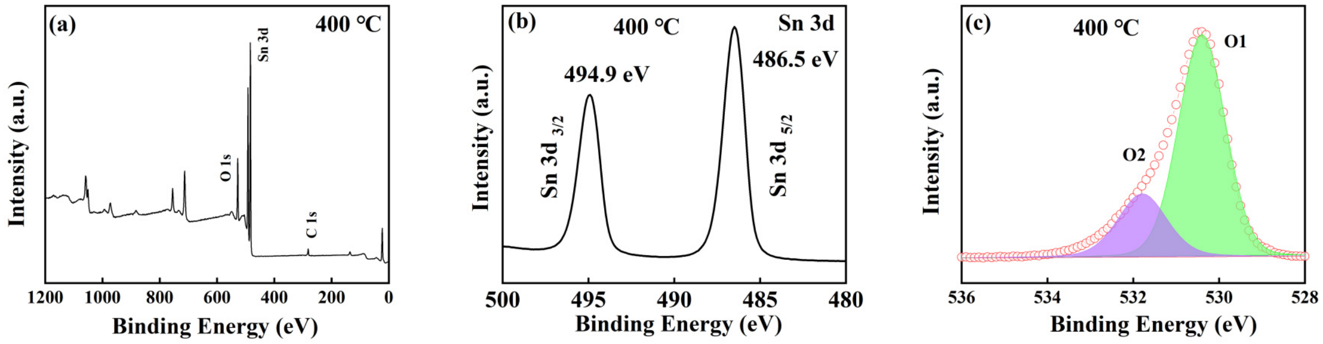

Figure S4. XPS spectra of SnO_2(400): (a) XPS full spectrum, (b)Sn 3d, (c) O1s.

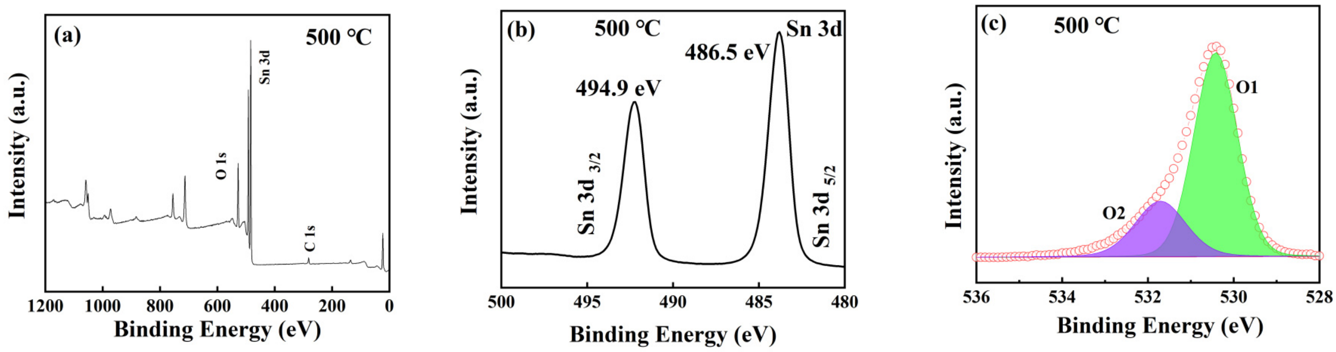

Figure S5. XPS spectra of SnO_2(500): (a) XPS full spectrum, (b)Sn 3d, (c) O1s.

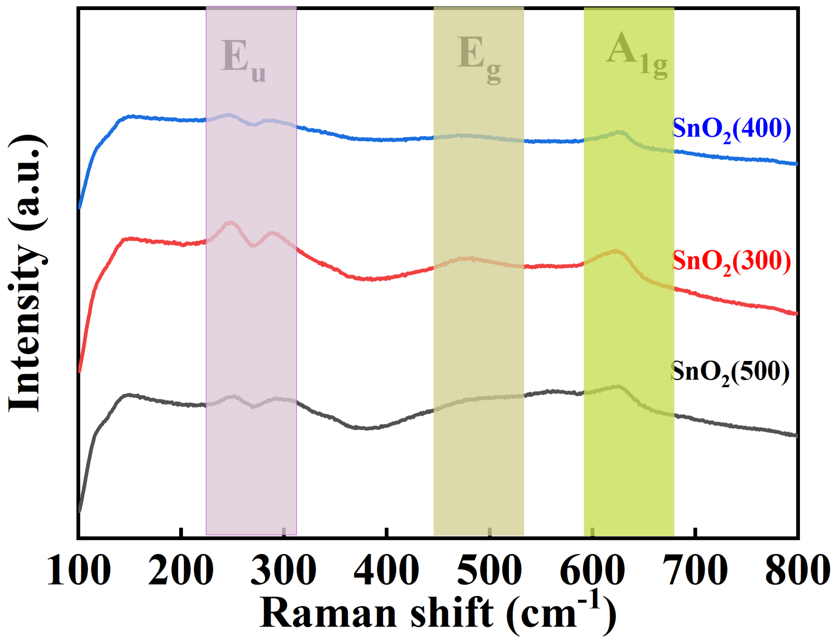

Figure S6. Raman spectra of SnO_2 samples

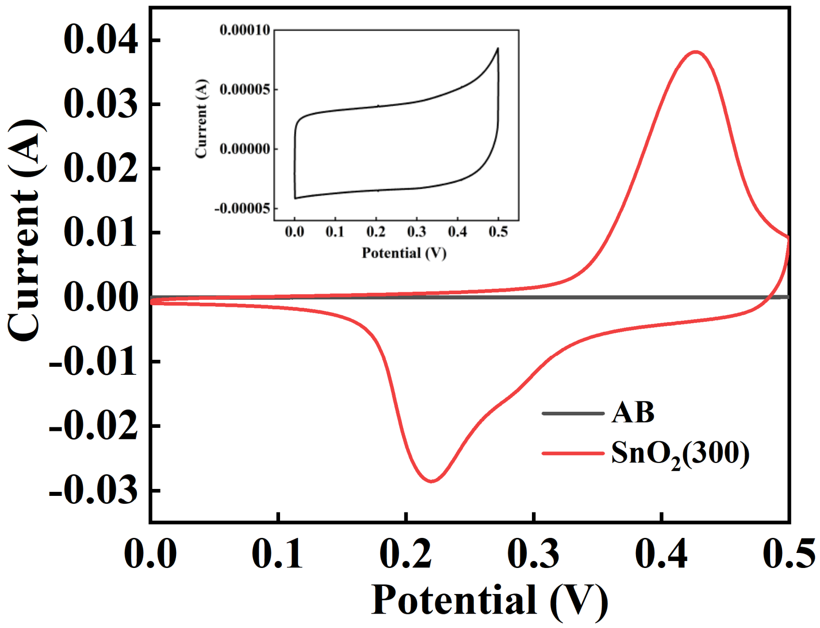

Figure S7. The CV curves of P-SnO_2(300) and AB at a scan rate of 50 mV s^{-1} in the three-electrode system.(Inset: CV of AB alone under identical conditions)

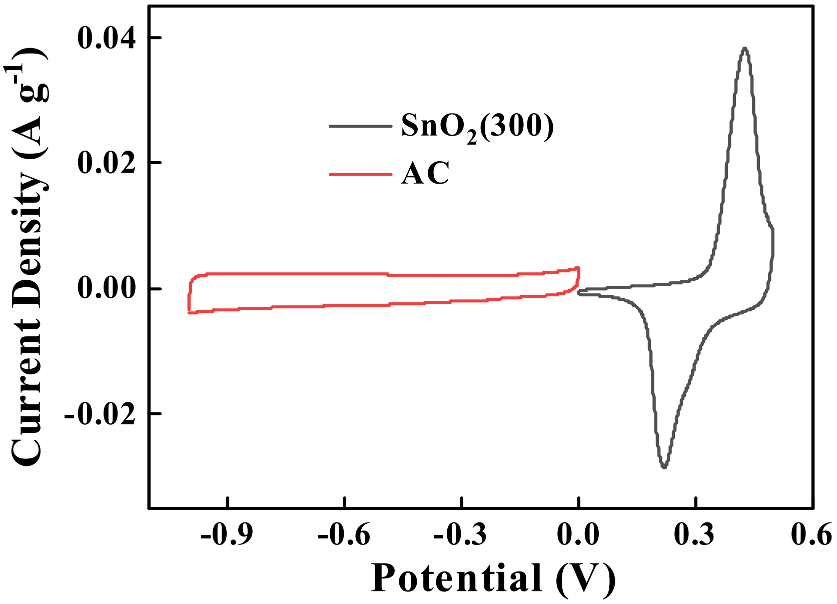

Figure S8. The CV curves of P-SnO_2(300)//AC measured at a scan rate of 50 mV s^{-1} in the three-electrode system.

Table S1. The synthetic methods, morphology, specific capacity (SC, 1 A g^{-1}), Sn source, theoretical yield (TY, 100% conversion to SnO_2), and actual yield (AY) of some SnO_2-based electrode materials.

Methods	Morphology	SC (F g^{-1})	Sn source (g or mol)	TY (g)	AY (g)	Refs.
Hydrothermal	Porous	241	$SnCl_2 \cdot 2H_2O$ (0.643 g)	0.4295	–	[1]
Hydrothermal	Nanosheet	247	$SnCl_2 \cdot 2H_2O$ (5 mmol)	0.7536	–	[2]
Hydrothermal	Nanocluster	197.7	$SnCl_4 \cdot 5H_2O$ (100 mg)	0.0430	–	[3]
Hydrothermal	Urchin-like	220	$SnCl_2 \cdot 2H_2O$ (0.45 g)	0.3306	–	[4]
Sol–gel	Snow-like	172	$SnCl_2 \cdot 2H_2O$ (0.85 g)	0.5677		[5]
Electrospinning	Nanowire	128.3	$SnCl_4 \cdot 5H_2O$ (96 mmol)	14.4682	–	[6]
Hydrothermal	2D flakes	234	$SnCl_2 \cdot 2H_2O$ (1 g)	0.6679	–	[7]
Hydrothermal	Microflower	217	$SnCl_2 \cdot 2H_2O$(1.1282 g)	0.7535	–	[8]
Hydrothermal	Microflower	252	$SnCl_2 \cdot 2H_2O$ (5 mmol)	0.7536	–	[9]
Hydrothermal	Microflower	205	$SnCl_2 \cdot 2H_2O$ (5 mmol)	0.7536	–	[10]
Co-precipitation	Spherical nanoparticles	183	$SnCl_2 \cdot 2H_2O$ (10 mmol)	1.5071		[11]
Thermal oxidation gas-release	Porous	267.31	$SnCl_2 \cdot 2H_2O$ 5.5625 g	3.7152	2.9232	This work
			$SnCl_2 \cdot 2H_2O$ 55.6250 g	37.1515	32.2472	
			$SnCl_2 \cdot 2H_2O$ 111.2500 g	74.3031	66.7579	

References

1. Guo, Y.D.; Abdu, H.I.; Hamouda, H.A.; Almamoun, O.; Aboudou, T.; Thani, E.S.; Radaki, S.A. Enhanced supercapacitor performance with hierarchically porous SnO_2 embedded in reduced graphene oxide nanosheets. *Mater. Res. Bull.* **2026**, *193*, 113662-113671.
2. Zhang, Y.D.; Hu, Z.A.; Liang, Y.R.; Yang, Y.Y.; An, N.; Li, Z.M.; Wu, H.Y. Growth of 3D SnO_2 nanosheets on carbon cloth as a binder-free electrode for supercapacitors. *J. Mater. Chem. A* **2015**, *3*, 15057-15067
3. Hong, X.D.; Li, S.L.; Wang, R.; Fu, J.W. Hierarchical SnO_2 nanoclusters wrapped functionalized carbonized cotton cloth for symmetrical supercapacitor. J. *Alloys Compd.* **2019**, *775*, 15-21.
4. Khan, A.; Ullah, I.; Khan, A.U.; Ahmad, B.; Katubi, K.M.; Alsaiari, N.S.; Saleem, M.; Ansari, M.Z.; Liu, J. Photocatalytic degradation and electrochemical energy storage properties of CuO/SnO_2 nanocomposites via the wet-chemical method. *Chemosphere* **2023**, *13*, 137482-137491.
5. Rafique, K.; Shah, M.Z.U.; Shah, A.; Shah, M.S.U.; Hou, H.Y.; Sajjad, M.; Arif, M.; Ahmad, S.A.; Hassan, N. Electrochemical performance evaluation of a newly developed $ZnS–SnO_2$ composite in an aqueous electrolyte. *J. Mater. Sci: Mater. Electron.* **2023**, *34*, 1717-1729.
6. Vijayan, B.L.; Krishnan, S.G.; Zain, N.K.M.; Harilal, M.; Yar, A.; Misnon, I.I.; Dennis, J.O.; Yusoff, M.M.; Jose, R. Large scale synthesis of binary composite nanowires in the Mn_2O_3-SnO_2 system with improved charge storage capabilities. *Chem. Eng. J.* **2017**, *327*, 962-972.
7. Anshu, S.; Priya, S.; Mandal, D.; Rahul, R.; Singh, T.; Chandra, A. 2D flakes of Au decorated SnO_2 nanoparticles as electrode material for high performing supercapacitor. *J. Phys. D: Appl. Phys.* **2023**, *56*, 205501-205516.
8. Ullah, E.; Shah, M.Z.U.; Ahmad, S.A.; Sajjad, M.; Khan, S.; Alzahrani, F.M.; Yahya, A.E.M.; Eldin, S.M.; Akkinepally, B.; Shah, A.; et al. Hydrothermal assisted synthesis of hierarchical SnO_2 micro flowers with CdO nanoparticles based membrane for energy storage applications. *Chemosphere* **2023**, *321*, 138004-138014.
9. Ullah, M.Z.; Shah, J.; Hayat, K.; Shah, S.K.; Hussain, I.; Khan, A.U.; Shah, M.S.; Hou, H.Y.; Sajjad, M.; Shah, A. *J. Energy Storage* **2025**, *175*, 109662-109669.
10. Muhammad M.Z.U.; Feng, J.; Shah A.; Sajjad, M.; Tirth, V.; Shah, M.S. Wet-chemical synthesis of SnO_2-Co_3O_4 microflower electrode for high energy density in aqueous asymmetric supercapacitors. *J. Energy Storage*, **2025**, *108*, 115081-115092.
11. Asaithambi, S.; Sakthivel, P.; Karuppaiah, M.; Sankar, G.U.; Balamurugan, K.; Yuvakkumar, R.; Thambidurai, M.; Ravi, G. Investigation of electrochemical properties of various transition metals doped SnO_2 spherical nanostructures for supercapacitor applications. *J. Energy Storage* **2020**, *31*, 101530-101541.
